# Tension-Enhanced Hydrogen Evolution Reaction on Vanadium Disulfide Monolayer

**DOI:** 10.1186/s11671-016-1329-5

**Published:** 2016-02-29

**Authors:** Hui Pan

**Affiliations:** Institute of Applied Physics and Materials Engineering, Faculty of Science and Technology, University of Macau, Macao SAR, China

**Keywords:** VS_2_ monolayer, Hydrogen evolution reduction, Water electrolysis, Tension, First-principles calculation

## Abstract

Water electrolysis is an efficient way for hydrogen production. Finding efficient, cheap, and eco-friendly electrocatalysts is essential to the development of this technology. In the work, we present a first-principles study on the effects of tension on the hydrogen evolution reaction of a novel electrocatalyst, vanadium disulfide (VS_2_) monolayer. Two electrocatalytic processes, individual and collective processes, are investigated. We show that the catalytic ability of VS_2_ monolayer at higher hydrogen coverage can be efficiently improved by escalating tension. We find that the individual process is easier to occur in a wide range of hydrogen coverage and the collective process is possible at a certain hydrogen coverage under the same tension. The best hydrogen evolution reaction with near-zero Gibbs free energy can be achieved by tuning tension. We further show that the change of catalytic activity with tension and hydrogen coverage is induced by the change of free carrier density around the Fermi level, that is, higher carrier density, better catalytic performance. It is expected that tension can be a simple way to improve the catalytic activity, leading to the design of novel electrocatalysts for efficient hydrogen production from water electrolysis.

## Background

Hydrogen, as an important energy carrier, is considered as a replacement of fossil fuels because of its abundant, clean, and renewable characteristics [1, 2]. Hydrogen production can be realized by photo-driven or electrical-driven water splitting [3–7], stream reforming [8], natural gas oxidization [9], carbonation [10], biomass electrolysis [11, 12], etc. Among these methods, catalytic water-spitting is highly appreciated because it is the cleanest way for hydrogen production, where hydrogen is evolving from proton to gas molecule by reduction [3–7]. Especially, electrical-driven water electrolysis has attracted increasing interests because of its high efficiency and eco-friendliness [7, 13–15]. To improve production efficiency and reduce cost, the design and fabrication of highly active, stable, and cheap electrocatalysts are critical to the development of green energy technology. It is well-known that noble metals and their alloys, such as platinum, are the most efficient catalysts in water electrolysis [16–22]. However, large-scale application of these catalysts in hydrogen evolution reaction (HER) for hydrogen production is difficult because they are rare and expensive. Alternatively, finding novel, environmental-friendly electrocatalysts with earth-abundant elements has been carried out with considerable efforts [7, 15].

Recently, transition metal dichalcogenides (TMDs) nanomaterials have been widely investigated as electrocatalysts in water electrolysis [23–32]. Particularly, The TMD monolayers with the formula of MX_2_, which has a sandwiched structure with one M-atom (M = transition metal element) layer enclosed within two X layers (X = chalcogen element), and the atoms in layers are hexagonally packed, have attracted increasing attention because of their high surface area, active edges states, and high mechanic flexibility [15, 25, 30–40]. For example, MX_2_ nanoribbons show high HER performance in water electrolysis due to their metallic edges [31], which can be further improved by edge doping and substrate [41, 42]. Metallic WS_2_ monolayers showed better HER performance than semiconducting counterpart [38]. Recently, Pan reported that VS_2_ monolayer shows excellent HER activity [32]. However, its performance is reduced at high hydrogen coverage because of reduced conductivity. To improve the HER performance of TMDs monolayers, the basic principle for the design of catalysts is to achieve a near-zero overpotential [18]. It has been reported that the improvement of HER activity can be achieved by doping, forming defect, and hybridizing with graphene [15, 23–42]. Voiry et al. [38] and Lee et al. [43] reported that mechanic strain can efficiently improve the HER activity of WS_2_ and MoS_2_ monolayers. To date, the effect of strain on the HER performance of TMDs monolayers at various hydrogen coverages and strength of tension has not been systematically studied. In this work, we carry out first-principles study on the improvement of the HER performance of VS_2_ monolayer with tension. We show that the improvement of catalytic activity of VS_2_ monolayer strongly depends to the strength of applied tension, hydrogen coverage, and reaction process. We find that the catalytic performance at higher hydrogen coverage can be enhanced by increasing tension, and both individual/collective processes can happen at the same tension depending on hydrogen coverage. We further show that the catalytic performance is closely related to free carrier density, which is controlled by the tension and hydrogen coverage.

## Methods

**Fig. 1:**
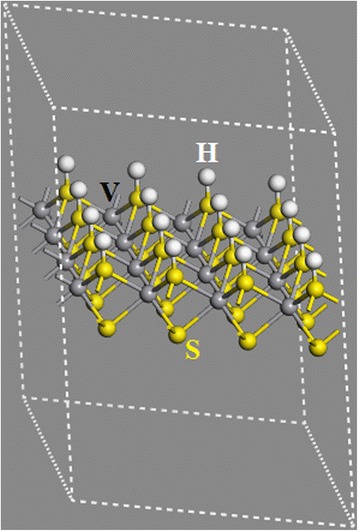
Atomic structure of VS_2_ monolayer with one surface fully covered by hydrogen atoms

First-principles calculations on the basis of the density functional theory (DFT) [44] and the Perdew-Burke-Eznerhof generalized gradient approximation (PBE-GGA) [45] are carried out to study the effect of tension on the HER activity of VS_2_ monolayer. The Vienna ab initio simulation package (VASP) [46] with projector augmented wave (PAW) scheme [47, 48] is used in our calculations. The Monkhorst and Pack scheme of k point sampling (3 × 3 × 1) is used for integration over the first Brillouin zone [49] and the cut-off energy is 450 eV. A sufficiently large supercell with 4 × 4 × 1 unit cells and a vacuum region of at least 20 Å in the vertical direction is used to investigate the hydrogen-coverage-dependent HER performance and avoid the interaction between images in neighboring cells (Fig. 1). Good convergence is obtained with these parameters and the total energy is converged to 2.0 × 10^−5^ eV/atom.

## Results and Discussion

The larger supercell with 4 × 4 × 1 unit cells is constructed based on the unit of VS2 monolayer with one surface fully covered by hydrogen atoms, that is, one S atom is bonded with one H atom on one side of the monolayer [50]. The hexagonal supercell with a lattice constant of 13.1 Å (a0) has 16 H, 32 S, and 16 V atoms (Fig. 1). To investigate the effect of tension on its HER performance, the lattice is statically extended from *a*_0_ to *a* by biaxial stretching or tension $\left(\varepsilon =\frac{a-{a}_0}{a_0}\times 100\ \%\right)$. The calculation on H-coverage-dependent HER performance is carried out by removing hydrogen atoms one by one from the surface. The H-coverage is defined as $\frac{n}{16}$ (*n* = 0 ~ 16). On the basis of the Sabatier principle, the HER performance of electrocatalyst in water electrolysis can be quantified by calculating the reaction free energy of hydrogen adsorption (ΔGH) [18, 19, 32, 33, 38], which can be obtained from following formula:

1 $$ \Delta {G}_{\mathrm{H}}=\Delta {E}_{\mathrm{H}}+\Delta {E}_{\mathrm{ZPE}}-T\Delta {S}_{\mathrm{H}} $$

where Δ*E*_H_ is the H-coverage-dependent hydrogen chemisorption energy. It can be differential chemisorption energy as calculated by:

2 $$ \Delta {E}_{\mathrm{H}}=E\left({\mathrm{VS}}_2+n\mathrm{H}\right)-E\left({\mathrm{VS}}_2+\left(n-1\right)\mathrm{H}\right)-\frac{1}{2}E\left({\mathrm{H}}_2\right) $$

or it can be average chemisorption energy calculated by

3 $$ \Delta {E}_{\mathrm{H}}=\left(E\left({\mathrm{VS}}_2+n\mathrm{H}\right)-E\left({\mathrm{VS}}_2\right)-\frac{n}{2}E\left({\mathrm{H}}_2\right)\right)/n $$

where *n* is the number of H atoms adsorbed on a VS_2_ monolayer under tension. The H-coverage-dependent ΔG_H_ can be obtained by changing *n. E*(VS_2_ *+ n*H), *E*(VS_2_), and *E*(H_2_) in Eqs. (2 and 3) are the energies of monolayer with hydrogen atoms (*n*), pure VS_2_ monolayer, and hydrogen molecule, respectively. Δ*S*_H_ is the difference in entropy. Δ*E*_ZPE_ is the difference in zero point energy between the adsorbed and the gas phase. *ΔE*_ZPE_ − *TΔS*_*H*_ is about 0.24 eV [18, 19]. Therefore, Eq. (1) can be simplified to *ΔG*_*H*_ = *ΔE*_*H*_ + 0.24. According to the two methods (Eqs. 2 and 3) for the calculation of hydrogen chemisorption energy, the reaction free energies can be defined as differential ΔG_H_ (d-ΔG_H_) and average ΔG_H_ (a-ΔG_H_), which can be used to express the hydrogen production in the individual and collective processes, respectively. The individual process describes that hydrogen is produced one by one, while the collective process shows that all of hydrogen atoms on the surface are simultaneously converted to molecules. It is required that electrocatalyst with optimal HER performance should have a ΔG_H_ near 0 eV.

**Fig. 2:**
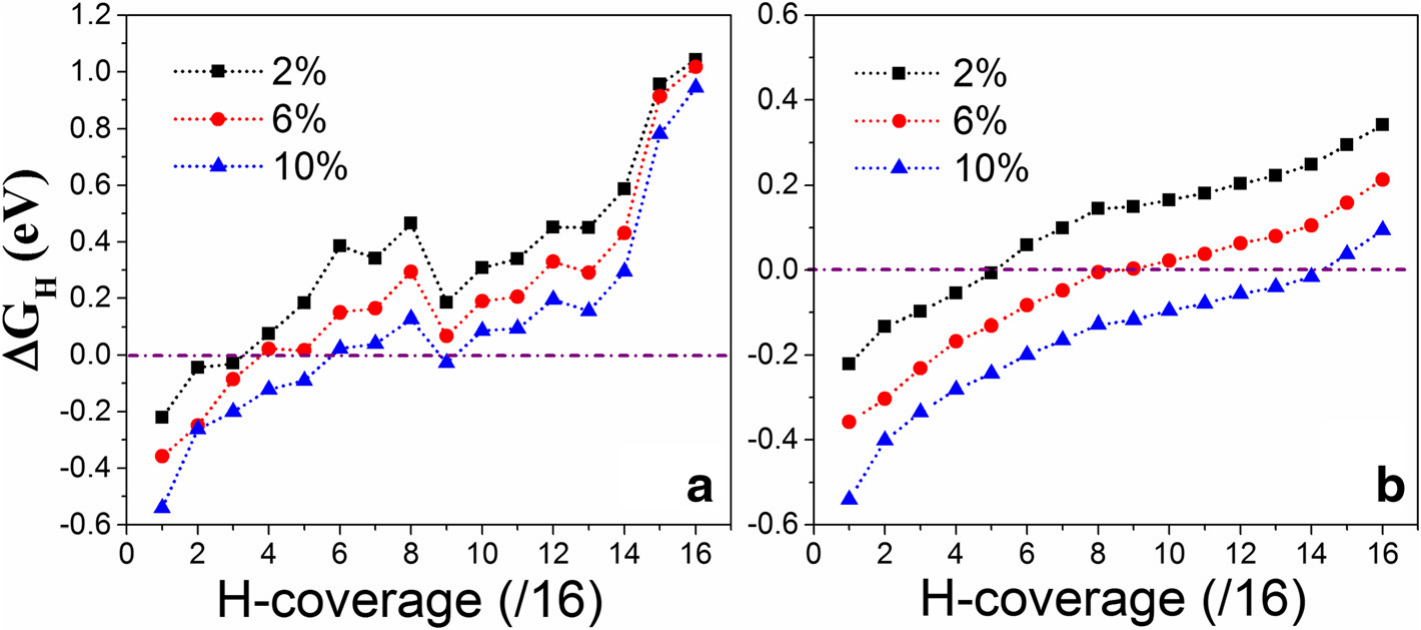
Calculated reaction free energy of hydrogen chemisorption as a function of hydrogen coverage: **a** differential free energy and **b** average free energy

Our first-principles calculations show that ΔG_H_ is dependent on the H-coverage and can be efficiently tuned by applied tension (Fig. 2). We see that the trend of d-ΔG_H_ as a function of hydrogen coverage under tension is almost similar to that without tension in the individual process (Δ*E*_H_ is calculated from Eq. 2), that is, d-ΔG_H_ increases as hydrogen coverage increases [32]. Importantly, d-ΔG_H_ is reduced upon the tension applied and the HER activity of VS_2_ monolayer in individual process at certain hydrogen coverage is improved by tension. The VS_2_ monolayer under *ɛ* = 2 % shows best HER performance in individual process with d-ΔG_H_ equal to −0.043 and −0.029 eV at the hydrogen coverages of $\frac{2}{16}$ and $\frac{3}{16}$, respectively (Fig. 2a). Under *ɛ* = 6 %, the best HER performance occurs at the hydrogen coverages of $\frac{4}{16}$ and $\frac{5}{16}$ with d-ΔG_H_ equal to 0.023 and 0.018 eV, respectively (Fig. 2a). Further increasing *ɛ* to 10 %, the VS_2_ monolayer shows best HER activity at the hydrogen coverages of $\frac{6}{16}$, $\frac{7}{16}$, and $\frac{9}{16}$ (d-ΔG_H_ = 0.024, 0.040, and −0.027 eV) (Fig. 2a). Clearly, the HER activity of VS_2_ monolayer in individual process is improved at relatively high H-coverage as tension increases, while that at low H-coverage is weakened. At the same time, we see that the effect of tension on the HER activity at full H-coverage $\left(\frac{16}{16}\right)$ is minor in the individual process (Fig. 2a), because removing one hydrogen atom is subjected to the strong attraction from the remained hydrogen atoms. Compared to the individual process, a-ΔG_H_ in the collective process (Δ*E*_H_ is calculated from Eq. 3) increases smoothly as the H-coverage increases (Fig. 2b). The best HER activity of VS_2_ monolayer with a-ΔG_H_ near zero (−0.007 eV) occurs at a hydrogen coverage of $\frac{5}{16}$ under *ɛ* = 2 %. Under *ɛ* = 6 %, the optimal performance (a-ΔG_H_ = −0.005~0.02 eV) is within a H-coverage range from $\frac{8}{16}$ to $\frac{10}{16}$. Further increasing tension to 10 %, VS_2_ monolayer is more active at a H-coverage of around $\frac{14}{16}$ (a-ΔG_H_ = −0.016 eV). Interestingly, ΔG_H_ in the collective process at full hydrogen coverage is significantly reduced upon the tension applied, which is about 0.1 eV/H at *ɛ* = 10 % (Fig. 2b). Although the absolute value of a-ΔG_H_ in the collective process is smaller than that in the individual process at full H-coverage, it should be difficult to happen if considering the total number of H atoms. By comparing the individual and collective processes (Fig. 2), we see that individual process is easier than collective process because absolute value of d-ΔG_H_ is smaller than that of a-ΔG_H_ multiplied by the number of H atoms on the surface of the monolayer. However, the collective process prefers to occur at certain H-coverage, where a-ΔG_H_ is close to zero (Fig. 2b). For example, if the VS_2_ monolayer is fully covered by hydrogen atoms $\left(\frac{16}{16}\right)$ initially at *ɛ* = 6 %, the individual process starts first. When the H-coverage is reduced to $\frac{9}{16}$ (a-ΔG_H_ = 0.003 eV), the collective process occurs because the total reaction energy in collective process (a-ΔG_H_ × 9 = 0.027 eV) is less than d-ΔG_H_ in individual process (0.07 eV). If the H-coverage on the VS_2_ monolayer is less than $\left(\frac{8}{16}\right)$, initially, only individual process will happen. Similarly, the collective processes under *ɛ* = 2 and 10 % are more easier than individual processes at hydrogen coverages of $\frac{5}{16}$ and $\frac{14}{16}$, respectively. Generally, the strained VS_2_ monolayer shows better catalytic performance than other TMDs monolayers and nanoribbons because of the near-zero Gibbs free energy in a wide range of hydrogen coverage.

**Fig. 3:**
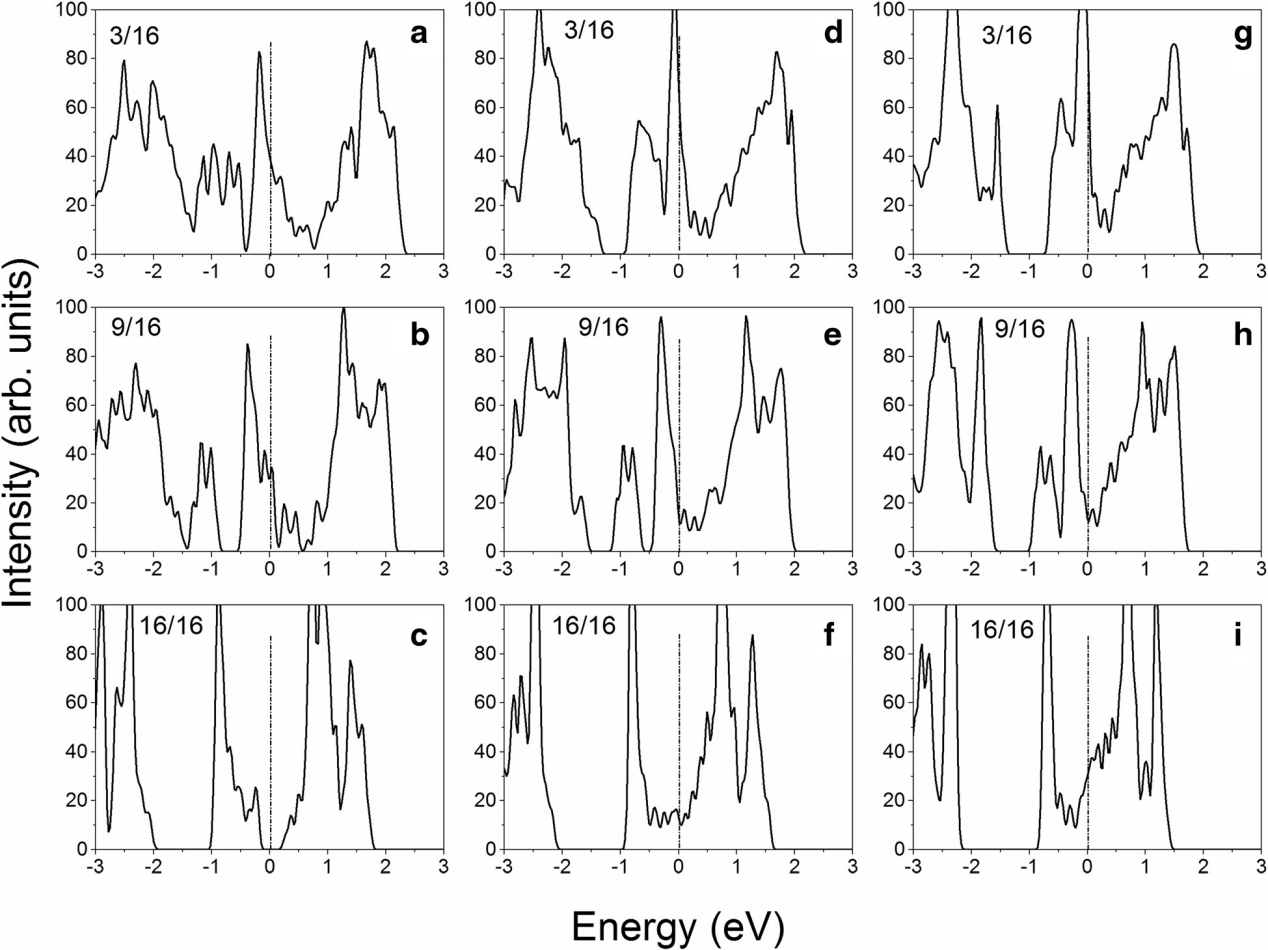
Calculated total density of states of VS_2_ monolayer under a tension of 2 % (**a**–**c**), 6 % (**d**–**f**), and 10 % (**g**–**i**) at a hydrogen coverage of 3/16, 9/16, and 16/16, respectively

To reveal the origin of H-coverage-dependent HER activity under tension, the density of state (DOS) is calculated. The carrier density can be estimated from the calculated DOSs. We see that the carrier density changes with the tension and hydrogen coverage (Fig. 3), which may indicate their effects on the HER activity in individual/collective process. At *ɛ* = 2 %, the VS_2_ monolayer shows strong metallic characteristic with high free carrier density under the Fermi level at a hydrogen coverage of $\frac{3}{16}$, as indicated by the continuous DOS spectrum from −3 to 2.2 eV (Fig. 3a). With the increase of hydrogen coverage to $\frac{9}{16}$, the DOS spectrum separates into two regions with a small gap from −0.9 to −0.5 eV, and the free carrier density under the Fermi level is accordingly reduced (Fig. 3b). Further increasing hydrogen coverage to $\frac{16}{16}$, the DOS spectrum separates into three regions and the Fermi level is within the band gap, resulting in semiconducting characteristic and poor free carrier density. The reduction of carrier density with the increment of hydrogen coverage should be responsible for the reduction of HER performance (Fig. 2a). At the same time, we see that the DOS spectrum of VS_2_ monolayer with a hydrogen coverage of $\frac{3}{16}$ under *ɛ* = 6 % is divided into two regions and the carrier density under the Fermi level is less than that under *ɛ* = 2 % (Fig. 3d), leading to the reduction of HER activity. Further increasing the tension (*ɛ* = 10 %), the gap between two DOS regions is enlarged and the area under the Fermi level is reduced, indicating reduced carrier density and resulting in further reduction of HER activity, which is consistent with the calculated Gibbs free energies (Fig. 2a). Comparing the DOSs of VS_2_ monolayers with a hydrogen coverage of 9/16 under *ɛ* = 2, 6, and 10 %, we see that the carrier density under the Fermi level increases as the tension increases (Figs. 3b, e, h), as indicated by the reduced/demolished gap around −0.5 eV, which is also consistent with the calculated Gibbs free energies (Fig. 2a). At full hydrogen coverage, we see that the HER activity of VS_2_ monolayer is not significantly improved (Fig. 2a). The calculated DOSs show that the Fermi levels are within conduction bands (Fig. 3f, i). However, the strong sharp DOS peaks around −0.8 eV may indicate localized states, which may reduce the carrier density and increase charge recombination centers, leading to differentially removing hydrogen difficult (Fig. 2a).

## Conclusions

We carry out first-principles to investigate the effects of tension on the hydrogen evolution reduction of VS_2_ monolayer. We find that tension can tune the HER performance of VS_2_ monolayer at different hydrogen coverage. The HER activity at high hydrogen coverage is greatly improved for both of individual and collective processes by increased tension. The hydrogen coverages for best HER performance in both individual and collective processes increase with the increment of tension. Generally, individual process is easier to occur in a wide range of hydrogen coverage, and collective process can happen at certain hydrogen coverage at the same tension. The optimal HER performance of VS_2_ monolayer with near-zero Gibbs free energy, comparable to Pt, can be achieved by tuning tension. We further show that the change of HER performance at different hydrogen coverages and under various tensions is closely related to the carrier density. High free carrier density is responsible for the improved HER activity. During revision, we noticed that Li et al. recently reported that strain can enhance the catalytic activity of MoS_2_ basal planes [51]. We see that the tension may provide an efficient way to modify the HER performance of VS_2_ and other 2D monolayers and find applications to water electrolysis for hydrogen production.
